# Birth Weight following Pregnancy during the 2003 Southern California Wildfires

**DOI:** 10.1289/ehp.1104515

**Published:** 2012-05-29

**Authors:** David M. Holstius, Colleen E. Reid, Bill M. Jesdale, Rachel Morello-Frosch

**Affiliations:** 1 School of Public Health, Environmental Health Sciences Division,; 2 Department of Environmental Science, Policy and Management, and; 3 School of Public Health, Community Health and Human Development, University of California, Berkeley, Berkeley, California, USA

**Keywords:** air pollution, birth weight, fetal growth retardation, fires, particulate matter, pregnancy outcomes

## Abstract

Background: In late October 2003, a series of wildfires exposed urban populations in Southern California to elevated levels of air pollution over several weeks. Previous research suggests that short-term hospital admissions for respiratory outcomes increased specifically as a result of these fires.

Objective: We assessed the impact of a wildfire event during pregnancy on birth weight among term infants.

Methods: Using records for singleton term births delivered to mothers residing in California’s South Coast Air Basin (SoCAB) during 2001–2005 (*n* = 886,034), we compared birth weights from pregnancies that took place entirely before or after the wildfire event (*n* = 747,590) with those where wildfires occurred during the first (*n* = 60,270), second (*n* = 39,435), or third (*n* = 38,739) trimester. The trimester-specific effects of wildfire exposure were estimated using a fixed-effects regression model with several maternal characteristics included as covariates.

Results: Compared with pregnancies before and after the wildfires, mean birth weight was estimated to be 7.0 g lower [95% confidence interval (CI): –11.8, –2.2] when the wildfire occurred during the third trimester, 9.7 g lower when it occurred during the second trimester (95% CI: –14.5, –4.8), and 3.3 g lower when it occurred during the first trimester (95% CI: –7.2, 0.6).

Conclusions: Pregnancy during the 2003 Southern California wildfires was associated with slightly reduced average birth weight among infants exposed *in utero*. The extent and increasing frequency of wildfire events may have implications for infant health and development.

In late October 2003, a series of wildfires burned > 750,000 acres of forest in Southern California (Blackwell and Tuttle 2003). Strong Santa Ana winds carried the resulting plumes of smoke toward Los Angeles and Orange counties, where a large urban population was exposed to elevated concentrations of air pollutants from the fires (Phuleria et al. 2005). An in-depth exposure assessment study estimated the population-weighted particulate matter (PM_10_; PM with aerodynamic diameter ≤ 10 µm) and fine particulate matter (PM_2.5_; PM with aerodynamic diameter ≤ 2.5 µm) concentrations, respectively, at 190 and 90 μg/m^3^ under heavy smoke conditions, and 125 and 75 μg/m^3^ under light smoke conditions, compared with baseline concentrations of 40 and 20 μg/m^3^ in the same region (Wu et al. 2006). Using that exposure assessment, a study (Delfino et al. 2009) of cardiorespiratory health effects estimated that elevated PM_2.5_ levels led to a 34% increase in hospital admissions for respiratory conditions 1–2 days later, with the largest associations observed among the very young (0–4 years, 8.3% per 10-μg/m^3^ increase in PM_2.5_) and very old (65–99 years, 10.1% per 10-μg/m^3^ increase in PM_2.5_); limited evidence supported a small increase in admissions for cardiovascular conditions as well (Delfino et al. 2009). A separate study found that parental recall of the smell of smoke during these fires was associated with increased medication usage, eye and respiratory symptoms, and physician visits among their children (Künzli et al. 2006).

*Health effects of wildfires*. Particulate matter (PM) is possibly the most important health-related component of wildfire events (Naeher et al. 2007). Wildfire-generated PM may be more toxic, on an equal-mass basis, than ambient PM collected in the same region during non-fire periods (Wegesser et al. 2009), potentially due to the role of atmospheric photochemistry resulting in the formation of secondary pollutants (Wegesser et al. 2010). Wildfires have been shown to enhance PM_2.5_ levels in many parts of the western United States (Jaffe et al. 2008), and recent studies have linked smoke exposure from wildfire events with spikes in morbidity in Canada (Henderson et al. 2011; Moore et al. 2006), Australia (Cameron et al. 2009; Chen et al. 2006; Johnston et al. 2002, 2007; Morgan et al. 2010; Tham et al. 2009), Southeast Asia (Emmanuel 2000; Mott et al. 2005; Sastry 2002), Finland (Hänninen et al. 2009) and California (Delfino et al. 2009; Künzli et al. 2006; Viswanathan et al. 2006); for a review, see Dennekamp and Abramson (2011).

Air pollution may not be the sole mechanism through which wildfire events affect health. For instance, wildfires threaten person and property; and news of an inherently unpredictable force of nature in itself may induce psychosocial stress in the population (Kumagai et al. 2004). Although the main effect of wildfire events on birth weight is likely mediated through their air pollution effects, distinguishing between these potential mechanisms is methodologically challenging.

*Objectives and study design*. Our objective was to estimate the birth weight effects associated with *in utero* exposure to a wildfire event. To our knowledge, to date one abstract has been published concerning the effects of wildfire smoke exposure on birth outcomes (Breton et al. 2011). Although little is known regarding the health effects of acute maternal exposures to smoke from wildfires, chronic maternal exposures to related hazards, including ambient particulate matter and indoor biomass smoke, have been linked to adverse birth outcomes, including lower birth weight. Many epidemiological studies have found associations between exposure to ambient PM and preterm birth or birth weight (for reviews, see Bosetti et al. 2010; Glinianaia et al. 2004). A recent meta-analysis of studies examining chronic maternal exposures to indoor air pollution in developing countries, such as that generated by cooking or heating with solid fuels, concluded that such exposures increase the risk of adverse pregnancy outcomes, including percent low birth weight, stillbirth, and reduced mean birth weight (Pope et al. 2010).

The study of natural experiments can be a methodologically useful way to advance research on air pollution and perinatal effects (Parker et al. 2008; Woodruff et al. 2009). Time-series studies can reduce threats to validity posed by exposure misclassification and confounding by variables that are associated with both ambient air pollution and perinatal outcomes, such as social class (Parker et al. 2008); thus they are a useful complement to observational studies of chronic exposures. With this in mind, we designed a time-series study to observe trimester-specific differences in mean birth weight among infants delivered to mothers residing in the South Coast Air Basin (SoCAB) before, during, and after the Southern California wildfires of 2003.

## Methods

*Study population*. We obtained birth records for infants delivered in the SoCAB from 1 January 2001 through 31 December 2005 from the Non-Confidential Birth Statistical Master File, provided by California’s Center for Health Statistics at the California Department of Health Services (California Automated Vital Statistics System 2006, unpublished data). We excluded preterm births (< 37 weeks gestation), post-term births (> 42 weeks gestation), and births with a reported birth weight < 1 kg or > 6 kg, yielding a total of 886,034 births for our analysis. Gestational ages were based on the number of days since the mother’s reported last menstrual period (LMP).

**Figure 1:**
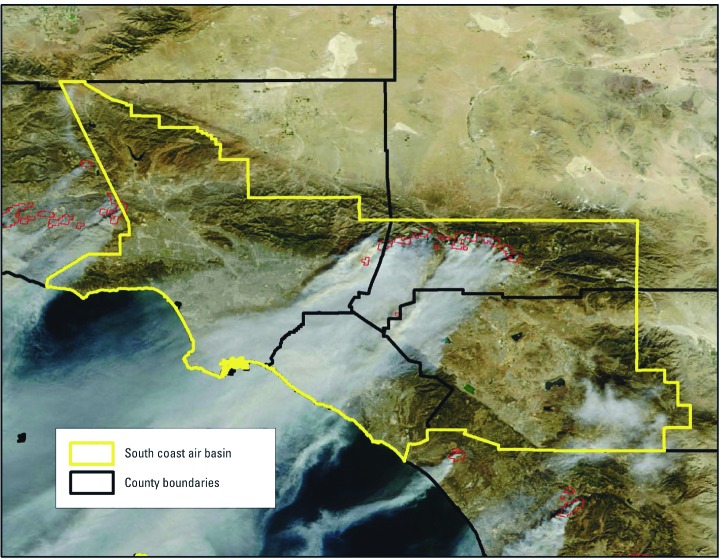
Geographic extent of the SoCAB study area, outlined in yellow, overlaid on MODIS satellite image from 26 October 2003. Active fires were outlined in red by NASA (NASA 2011).

The SoCAB, which forms the geographic basis for our study population, includes the entirety of Orange County as well as populous areas within Los Angeles, San Bernardino, and Riverside counties. This air basin was chosen as the boundary for the study on the basis of satellite images of the fires; it is partially bounded by mountains that trap air pollution in the absence of wind (Figure 1). The SoCAB does not contain Ventura and San Diego counties, which were also exposed to smoke during the 2003 wildfires.

*Exposure assessment*. On the basis of reports from the California Department of Forestry and Fire Protection (Blackwell and Tuttle 2003) and inspection of Moderate Resolution Imaging Spectroradiometer (MODIS) satellite imagery (NASA 2011), we defined the window of potential wildfire exposure as 21 October–10 November 2003. Most births in our study population (*n* = 491,496) were delivered before 21 October 2003 and could not have been exposed *in utero*. An additional 256,094 births were assigned an LMP later than 10 November 2003, and were also classified as unexposed. All remaining births (*n* = 138,444) were classified as exposed on the basis of temporal overlap between the wildfire exposure window and gestational intervals.

Our primary analysis used this temporal contrast as the basis for exposure assessment. However, we also conducted a sensitivity analysis in which we examined spatial contrasts based on proximity of maternal residence census tracts to air monitors. Tracts closer to monitors with average PM_10_ measures during the fires of < 40 µg/m^3^ were classified as low exposure, and tracts with average daily levels > 40 µg/m^3^ were classified as high exposure. This cut point split the PM_10_ monitors in the SoCAB in half, with 36% of births that gestated during the fires occurring in high exposure census tracts.

**Figure 2:**
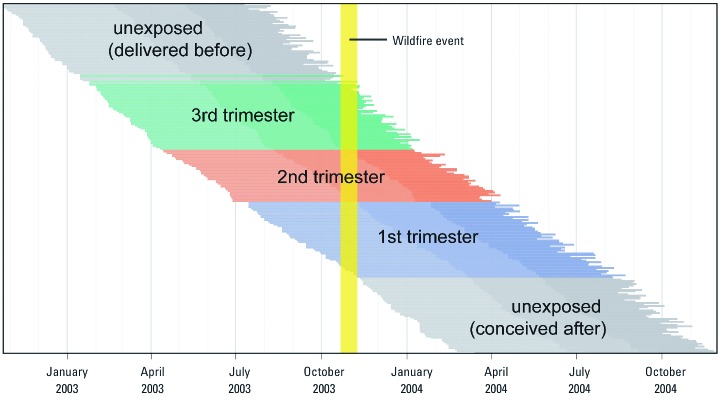
Schematic illustrating exposure assignment. Exposure status was assigned based on the overlap between the wildfire event (yellow) and estimated gestational intervals (horizontal segments). For clarity, gestational intervals are shown ordered from top to bottom by the LMP, and only a 0.1% sample from 2002–2004 is shown. Dates on the *x*-axis correspond to the beginning of quarters used to adjust for seasonality.

*Covariates and primary model*. We fit the data to a linear fixed-effects model (Equation 1) with birth weight (*y_i_*) as a continuous outcome and *x_ij_* as an indicator of exposure for birth *i* in trimester *j*. For our primary analysis, we defined *x_i_* as a categorical variable with four levels: exposed in trimester 1; exposed in trimester 2; exposed in trimester 3; or unexposed (Figure 2). We defined the first trimester as weeks 1–16 since LMP; the second as weeks 17–28; and the third as week 29 through the end of gestation. When the wildfire event overlapped with two trimesters, we assigned exposure to the trimester with the greater number of days of overlap. We controlled for maternal and birth characteristics (*z_i_*) associated with birth weight and included terms based on the date of the LMP (*t_i_*) to control for seasonality and trend.

*y_i_ =* β*_0_* + β*_1_x_i1_* + β*_2_x_i2_* + β*_3_x_i3_* + η*´z_i_* + *f*(*t_i_*) + ε*_i_* [1]

Our analysis was limited by the availability of information on administrative birth records. Among the variables included on these forms, we adjusted for maternal characteristics (age, educational attainment, parity, race/ethnicity) and characteristics of the birth itself (infant’s sex, gestational age) known to have a substantial influence on birth weight. These variables may have confounding potential, or play a role in explaining variation even in the absence of a confounding effect. We included parity of the mother with three levels: first live birth (reference), second live birth, or third or more live births. We coded gestational age (weeks since LMP) as 37 (reference), 38, 39, 40, 41, or 42 weeks. As a proxy for socioeconomic status, we coded maternal education with four levels: less than a high school education; completed high school or equivalent (reference); 1–3 years of postsecondary education; and ≥ 4 years of postsecondary education. We coded maternal race/ethnicity as non-Hispanic white (reference); Hispanic of any race; non-Hispanic black; non-Hispanic Asian; and unknown/multiple/other. We also included fetal sex with male as the referent category.

To account for secular trend and seasonal effects, we parameterized *f*(*t_i_*) as a combination of a linear secular trend based on the date of the last menstrual period for birth *i* (*t_i_*) and categorical indicator terms for the season of birth (in quartiles): Q1 (January–March, reference), Q2 (April–June), Q3 (July–September), or Q4 (October–December):

*f*(*t_i_*) = β*_trend_*(*t_i_*) + β*_Q2_I_Q2_* + β*_Q3_I_Q3_* + β*_Q4_I_Q4_* [2]

*Sensitivity analyses*. In addition to our primary analyses, we conducted three sensitivity analyses. In the first, we dichotomized the population according to whether the maternal residence census tract was closer to a PM_10_ monitor active during the fire period with an average PM_10_ measure > 40 μg/m^3^, or closer to a PM_10_ monitor with a lower average PM_10_ during the fire period. In the second, because of the association between season and trimester of exposure (Table 1, Figure 2), we instead parameterized *f*(*t_i_*) as a smooth, periodic, sinusoidal function of time known as the cosinor [Barnett and Dobson 2010; see Supplemental Material, Equations S1–S4 (http://dx.doi.org/10.1289/ehp.1104515)]. In the third, because accounts differ concerning the length of the wildfire event, we reduced the length of the wildfire event to peak exposure periods of 2 weeks or 1 week instead of 3 (keeping the starting date unchanged) and reassigned exposures accordingly.

**Table 1:** Maternal and infant characteristics (%), by wildfire event exposure status and trimester of exposure—SoCAB (*n* = 886,034).

Variable	Unexposed (n = 747,590)	Exposed by trimester (n = 138,444)				
		First (n = 60,270)	Second (n = 39,435)	Third (n = 38,739)
Fetal sex								
Male		51.0		51.0		50.7		50.9
Female		49.0		49.0		49.3		49.1
Gestational age (weeks)								
37		4.8		4.7		5.0		3.9
38		10.9		10.6		11.1		9.7
39		23.0		22.6		22.9		21.9
40		29.2		29.9		29.5		29.5
41		22.6		22.6		21.9		24.1
42		9.4		9.6		9.6		10.9
Parity								
1		38.1		38.7		38.8		39.5
2		31.8		31.8		31.5		31.3
3 or more		30.1		29.5		29.7		29.1
Maternal age (years)								
< 18		3.1		3.0		3.0		3.0
18–34		76.1		75.8		76.0		76.4
35–50		20.8		21.2		21.0		20.5
Maternal education								
Less than high school		32.3		31.0		31.7		31.9
Completed high school or equivalent		28.4		27.6		27.8		28.6
1–3 years postsecondary		18.1		18.5		17.8		17.9
≥ 4 years postsecondary		21.2		22.9		22.7		21.5
Maternal race/ethnicity								
Hispanic		60.6		60.0		59.9		60.5
Non-Hispanic white		22.0		22.6		22.3		21.3
Non-Hispanic Asian		11.2		11.4		11.6		11.9
Non-Hispanic black		5.9		5.7		5.9		6.0
Non-Hispanic other/unknown		0.3		0.3		0.3		0.3
Season								
Q1 (January–March)		24.8		0.0		0.0		87.6
Q2 (April–June)		23.0		0.0		94.8		12.4
Q3 (July–September)		23.0		66.7		5.2		0.0
Q4 (October–December)		29.3		33.3		0.0		0.0

*Statistical software*. We used R version 2.14.0 and the stats::lm() function for model fitting. To fit the cosinor-based seasonal model, we used the season package, version 0.2–6 (R Project for Statistical Computing, Vienna, Austria).

## Results

Of the 886,034 births in our analysis, 84.4% (*n* = 747,590) were unexposed *in utero*. Of the 138,444 exposed, 28.0% (*n* = 38,739) were exposed in the third trimester, 28.5% (*n* = 39,435) were exposed in the second trimester, and 43.5% (*n* = 60,270) were exposed in the first trimester (Table 1).

Most infants in our study were delivered to Hispanic mothers (60.5%), followed by non-Hispanic white (22.0%), non-Hispanic Asian or Pacific Islander (11.3%), and non-Hispanic black (5.9%) mothers. Most infants were delivered to mothers 18–34 years of age (76.1%), with 20.8% delivered to mothers 35–50 years of age and 3.1% delivered to mothers 15–18 years of age. Approximately one-third of mothers had less than a high school education (32.1%), whereas 28.4% had completed a high school degree or equivalent, 18.1% had 1–3 years of postsecondary education, and 21.4% had ≥ 4 years of postsecondary education.

We did not observe substantive differences between the exposed and unexposed with respect to measured covariates, except for season of birth (Table 1). Effect estimates for covariates are reported in Supplemental Material, Table S1 (http://dx.doi.org/10.1289/ehp.1104515).

*Estimated effects of wildfire exposure.* Adjusted models revealed that mean birth weight was 6.1 g lower [95% confidence interval (CI): –8.7, –3.5] among infants exposed *in utero* during any trimester compared with unexposed infants [see Supplemental Material, Table S1 (http://dx.doi.org/10.1289/ehp.1104515)]. Among those exposed in the third trimester, we observed a reduction of 7.0 g (95% CI: –11.8, –2.2). The largest estimated effect was observed in the second trimester, with a reduction of 9.7 g (95% CI: –14.5, –4.8). Among infants exposed in the first trimester, we also estimated a decline in mean birth weight but it was not statistically significant (3.3 g; 95% CI: –7.2, 0.6) (Table 2). Both unadjusted and adjusted estimates are reported in Table 2; subsequent discussion is restricted to the adjusted model. We also restricted the peak exposure period by specifying models with the wildfire duration defined as 2 weeks or 1 week of peak intensity instead of 3 weeks to assess sensitivity to the duration of the wildfire event, but main effects were not substantively altered (data not shown).

**Table 2:** Estimated effect of wildfire event during gestation on birth weight (g), by trimester.

Unadjusted model			Adjusted model		
Trimester of exposure	Effect (g)	95% CI	Effect (g)	95% CI
Third (≥ 29 weeks)		–7.9		(–12.8, –3.1)		–7.0		(–11.8, –2.2)
Second (17–28 weeks)		–17.1		(–21.9, –12.3)		–9.7		(–14.5, –4.8)
First (1–16 weeks)		–3.9		(–7.8, 0.0)		–3.3		(–7.2, 0.6)
Any trimester		–8.8		(–11.5, –6.1)		–6.1		(–8.7, –3.5)
Adjusted model includes terms for fetal sex, gestational age, parity, maternal age, maternal education, maternal race/ethnicity, secular trend, and season.								

Among births in census tracts proximal to monitors with higher average PM_10_ during the wildfire event, the estimated decrement in birth weight associated with pregnancy (any trimester) during the event was 6.6 g (95% CI: –11.0, –2.2). In tracts more proximal to monitors with average PM_10_ levels < 40 μg/m^3^, the estimated decrement in birth weight associated with pregnancy during the event was 5.9 g (95% CI: –9.2, –2.6). These estimates were not discernibly different from each other [see Supplemental Material, Table S3 (http://dx.doi.org/10.1289/ehp.1104515)].

*Seasonality and trend*. Over the entire period, 2001–2005, we found a secular decline in mean birth weight of 6.9 g/year (95% CI: –7.6, –6.2) [see Supplemental Material, Table S1 (http://dx.doi.org/10.1289/ehp.1104515)]. Those conceived in Q3 (July–September) had the highest estimated mean weight at birth, 11.9 g (95% CI: 9.0, 14.7) more than infants conceived in Q1 (January–March), the referent time period. Infants conceived in Q1 weighed the least.

When we conducted a sensitivity analysis, using an alternate model with seasonal effects parameterized as a cosinor, the magnitude of the seasonal effect was comparable (11.6 g; 95% CI: 7.7, 15.5), as was the relative timing (i.e., phase) of highest birth weight, with the maximum occurring on 4 August (95% CI: 29 July, 7 August) [see Supplemental Material, Table S2 (http://dx.doi.org/10.1289/ehp.1104515)].

*Goodness-of-fit and residuals*. The adjusted *R*^2^ for the full model was 0.109. We plotted residuals versus fitted values and constructed a quantile probability plot to verify that the residual distribution was normal. We did not observe any heteroskedasticity of the residuals (data not shown).

## Discussion

We observed a slight reduction in estimated mean birth weight among term infants exposed *in utero* to the 2003 California wildfires. The strongest estimated effect was observed for second-trimester exposure, followed by third-trimester exposure.

Climate change scientists predict that wildfires will increase in frequency and magnitude as global temperatures increase and rainfall patterns change (Westerling and Bryant 2008; Westerling et al. 2006). These increases in wildfire events are projected to add to atmospheric pollution in the western United States under various climate change scenarios (Spracklen et al. 2009). In California, smoke impacts are already a required consideration in the planning and execution of preventive wildfire management activities, such as prescribed burns. For example, forest management professionals are required to assess the likely direction of smoke plumes and gauge their potential for impact on smoke sensitive areas (State of California 2001). Kochi et al. (2010) make the case that optimal wildfire management policy should explicitly include estimates of health-related and economic costs of wildfire smoke exposure.

*Potential etiologic pathways.* At least two categories of etiologic pathways plausibly link maternal wildfire exposure with lower birth weight: biological (exposure to air pollution from the fires) and psychosocial (stress caused by direct or indirect consequences of wildfires). A combination of the two is also plausible. We cannot differentiate the contributions of these two pathways here, because we were not able to quantify daily air pollution exposures for each birth, or individual or ecological indicators of maternal stress. Nevertheless, our results may reflect the potential conjoint effect of these two pathways.

Among the biological mechanisms hypothesized as having a possible effect on intrauterine growth rate are hypoxia and/or oxidative stress resulting from exposure to woodsmoke constituents, including carbon monoxide and PM (Siddiqui et al. 2008), alteration of maternal–placental exchanges, endocrine disruption, and oxidative stress pathways leading to alteration of maternal host–defense mechanisms and subsequently higher risk of infections (Slama et al. 2008). Reviews on the topic have found limited applicable research from animal and toxicological studies to distinguish these possible mechanisms (Ritz and Wilhelm 2008; Slama et al. 2008; Woodruff et al. 2009). Human studies of the acute effects of wildfire smoke exposure on firefighters have demonstrated inflammatory responses and pulmonary function test declines (e.g., Swiston et al. 2008). Human experiments in which healthy nonsmokers were exposed to woodsmoke under controlled conditions, with concentrations of PM_2.5_ > 240 µg/m^3^ for up to 4 hr, resulted in elevated levels of blood and urine biomarkers indicating oxidative stress and pulmonary inflammation in the lower airways (Barregard et al. 2006, 2008; Sällsten et al. 2006). More recent experiments confirmed biomarkers of systemic and pulmonary inflammation in blood and lavage, but found no effect on pulmonary function or self-reported symptoms, and minimal effects on indices of heart rate variability (Ghio et al. 2012).

Psychosocial aspects of wildfire exposure may also contribute to adverse health outcomes, although this is an understudied topic (Kumagai et al. 2004). Several studies have observed signs of fetal stress and adverse birth outcomes in the aftermath of disasters such as earthquakes (Weissman et al. 1989), shipwrecks (Catalano and Hartig 2001), and terrorist attacks (Catalano et al. 2005). Plausible causes of stress in the wake of wildfires include loss of property, shelter, money, and other basic individual resources; physical incapacitation or injury; and disruption of sharing and support networks (Fowler 2003).

Further analyses with methods that better characterize individual-level pollution exposures and psychosocial stress would be required to distinguish the relative contribution of the two pathways.

*Exposure misclassification.* Our study captured temporal variation in wildfire exposure, but was limited in its ability to account for spatial variation. The SoCAB includes areas that were likely not directly exposed to heavy smoke plumes or to significant concentrations of diffused smoke. Because we relied on administrative vital statistics records, we were not able to assess whether mothers resided within the air basin throughout their pregnancies, or determine how much time they spent at their primary residence. Meteorology, time–activity patterns, and variations in the built environment likely all contributed to variations in individual exposures, and therefore to exposure misclassification. Because our study did not capture variations in exposure among the exposed, we could not evaluate dose–response relationships. A relatively small number of highly exposed mothers in this region may have been affected to a greater degree than our estimates would predict.

When we conducted a sensitivity analysis that distinguished between births located in higher versus lower PM_10_ tracts during the wildfire event, we observed that the decrease in birth weight associated with gestational wildfire exposure was similar between these two populations. This result may be attributable to the fact that monitoring results could not adequately capture differences in ambient PM_10_ levels. Improved analysis with PM_10_ as a continuous variable or modeling of the PM exposure using satellite data or chemical transport models might reveal a relationship between wildfire-related PM exposure and birth weight. Alternatively, it is also possible that the associations with decreased birth weight were mediated not by air pollution but by some other mechanism, such as stress.

Several factors could explain our finding of stronger associations with exposure during the second and third trimesters than exposure during the first trimester. First, there is the issue of exposure misclassification. Given that the date of conception is less certain than the date of delivery, it is possible that some infants were categorized as exposed in the first trimester, when in fact their conception date occurred after the fire was over. This misclassification bias is unlikely to affect exposure assessment in the third or second trimester, but may lead to an underestimate of effects during the first trimester. Overestimation of the length of the wildfire event would also have resulted in some unexposed births being misclassified as first-trimester exposures. However, reducing the length of the wildfire event did not substantively alter our main effects.

Exposure could also have increased the risk of preterm birth or fetal loss. Because we excluded preterm births and fetal losses, excess preterm delivery or fetal loss among the first-trimester exposed could have differentially eliminated the most vulnerable from our sample. When Breton et al. (2011) examined prenatal exposure to high PM_2.5_ levels from the same wildfires among eight counties in Southern California, using vital statistics records for 2003–2004, they did not estimate a significant effect on preterm birth, but they also did not assess fetal loss. The effects of wildfire exposure on birth weight could also be stronger among those exposed in the second or third trimesters for reasons that are not yet understood. Further examination of the effects of trimester-specific exposures in other studies may help to resolve this question.

*Seasonal confounding.* Controlling for seasonal variation in time-series studies of air pollution can be a challenge (Slama et al. 2008; Woodruff et al. 2009). For example, both temperature and ambient (non-wildfire) air pollution exhibit seasonal patterns, and these patterns themselves vary geographically due to differences in regional characteristics. When we controlled for seasonality using quarterly indicators, 87.6% of the infants exposed in the third trimester were conceived in Q1 (January–March), and 94.8% of those exposed in the second trimester were conceived in Q2 (April–June) (Table 1, Figure 2). This raised the possibility of confounding between trimester-specific wildfire exposure and conception in the first half of the year.

To address this, we conducted a sensitivity analysis, parameterizing the seasonal component of the model as a smooth, continuous, and periodic function of time: the cosinor (Barnett and Dobson 2010). The general form of the cosinor is sinusoidal, like many natural seasonal phenomena, and has only two degrees of freedom, amplitude and phase [see Supplemental Material, Equations S1–S4 (http://dx.doi.org/10.1289/ehp.1104515)]. As such, it is readily interpretable, and it has been widely applied to the analysis of seasonal and circadian rhythms (Barnett and Dobson 2010).

The cosinor-based analysis yielded effect estimates consistent with the pattern described by our primary model, increasing our confidence in the results. The peak-to-peak amplitude (11.6 g) in seasonal variation was similar to the difference between the minimum and maximum seasonal coefficients from the model using indicator terms (11.9 g). The phase was also consistent with the primary model’s seasons of lowest and highest average birth weight [January–March (Q1) and July–September (Q3), respectively].

*Other potential confounders.* We adjusted for several individual-level covariates known to be associated with birth weight, but data on other potential confounders were not available. For example, maternal smoking is not reported on most California birth records, and its inclusion in our study may have changed our results. However, recent studies suggest that although smoking during pregnancy has a large effect on birth weight, in studies of ambient air pollution it does not significantly confound the association between ambient air pollution exposure and adverse perinatal outcomes such as infant mortality and preterm birth (Basu et al. 2003; Darrow et al. 2006).

In previous research of wildfire health effects, few studies have attempted to separate the fraction of smoke attributable to wildfire from that attributable to background air pollution (for a review, see Dennekamp and Abramson 2011). In areas with significant sources of other pollution, such as the SoCAB, apportionment can be a challenge. Observations from the nearest monitor, which are often used to characterize background air pollution, can be missing during a wildfire episode, sometimes due to the fire itself. To obtain ecologic or individual-level estimates of smoke exposure, several methods can be employed: satellite imagery, dispersion or chemical transport modeling, and/or spatiotemporal interpolation. However, each of these has associated difficulties in implementation and interpretation, especially during a short time window with atypical meteorology, such as the strong Santa Ana winds that fanned the 2003 fires.

To the extent that variation in wildfire-attributable pollution and background pollution are independent, including background pollution in the model could improve the precision of effect estimates, but should not affect the central tendencies. On the other hand, insofar as background concentrations are correlated in space or time with wildfire smoke concentrations (e.g., to the extent that they are similarly determined by physical geography), including background pollution could induce confounding just as including seasonality can. Without access to detailed measurements of both fractions, we elected to consider a strictly temporal contrast, reserving spatiotemporal refinements of exposure for future work.

Previous studies that have attempted to isolate the contribution of wildfire-generated smoke have also compared health effects to a reference period (e.g., Delfino et al. 2009). However, in any interrupted time-series study, there is always the possibility of an unmeasured confounder with a similar temporal profile to that of the exposure. For example, if a foodborne illness outbreak happened at the same time as the wildfires, and had a negative impact on birth weight, it could conceivably explain part or all of the observed effect. The fact that our unexposed births were drawn from both before and after the exposure window, and from other years at the same time of year, helps to reduce such threats to validity, but cannot eliminate them.

## Conclusion

This study indicates that maternal exposure to wildfire events may result in modestly lower infant birth weight. A small decline in birth weight is unlikely to have clinical relevance for individual infants, and there is debate about whether a small shift in the population distribution of birth weight has broader health implications (e.g., Wilcox 2001). Although the effects we estimated are much smaller than for many other exposures, such as smoking, the extent of exposures during wildfire events and their increasing frequency suggests potentially important implications for infant health and development. Finally, future research should also assess alternative mechanistic pathways besides air pollution (such as stress) for understanding the adverse health effects of wildfire events.

## Supplemental Material

(152 KB) PDF
