# Supplementary material for: Birth Weight following Pregnancy during the 2003 Southern California Wildfires
Source: Environ Health Perspect. 2012 May 29;120(9):1340–5. doi: 10.1289/ehp.1104515 (PMC3440113; doi:10.1289/ehp.1104515)
Supplement: (152 KB) PDF [file ehp.1104515.s001.pdf]

Supplemental Material

Manuscript Title: Birth Weight Following Pregnancy During the 2003 Southern California Wildfires

Authors: David M. Holstius,[1] Colleen E. Reid,[1] Bill M. Jesdale,[2] Rachel Morello-Frosch[2,3]

Author Affiliations:

[1] School of Public Health, Environmental Health Sciences Division, University of California, Berkeley, CA

[2] Department of Environmental Science, Policy and Management, University of California, Berkeley, CA

[3]School of Public Health, Community Health and Human Development, University of California, Berkeley, CA

Corresponding Author:

Rachel Morello-Frosch

Department of Environmental Science Policy and Management

and School of Public Health

137 Mulford Hall, University of California

Berkeley, CA 94720-3114

rmf@berkeley.edu
Phone: 510-643-6348

FAX: 510-643-5438

Contents for Supplementary Material

Page 3. Supplemental Material; Modeling seasonality with a cosinor (text)

Page 4. Supplemental Material, Table S1. Linear model results for birth weight, in grams, showing effect estimates for all covariates, exposed during any trimester, and each trimester individually, vs. all trimesters outside fire period.

Page 5. Supplemental Material, Table S2. Linear model results for birth weight, in grams, showing effect estimates for all covariates, fire period during each trimester individually vs. all trimesters outside fire period (using cosinor approach rather than seasonal indicator variables).

Page 6. Supplemental Material, Table S3. Linear model results for birth weight, in grams, showing effect estimates for all covariates, fire period during any trimester vs. all trimesters outside fire period, stratified by proximity to monitors with highest PM measures during fire event.

Modeling Seasonality with a Cosinor

Under the cosinor-based approach, the temporal term $\boldsymbol{f}(t_i)$ is constructed from a modified cosine function having phase P, frequency ω, and amplitude A:

$$\boldsymbol{f}(t_i) = \alpha_0 t_i + \mathrm{A}\cos(\omega t_i - P) \qquad \text{[S1]}$$

The additive term $\alpha_0 t_i$ models the secular trend, since t_i represents the LMP for birth i. The frequency ω is chosen according to the length of the modeled cycle; in this case, we are interested in modeling an annual cycle, so $\omega \approx 2\pi$ / 365.14 days^{-1}. The following expression is equivalent to Eq (1), but is linear in t_i, and so can be fit using standard techniques such as least squares:

$$\boldsymbol{f}(t_i) = \alpha_0 t_i + c\cos(\omega t_i) + s\sin(\omega t_i) \qquad \text{[S2]}$$

In terms of the coefficients c and s, the amplitude A then corresponds to:

$$\mathrm{A} = \sqrt{\mathrm{c}^2 + \mathrm{s}^2} \qquad \text{[S3]}$$

While the phase P (in radians) corresponds to:

$$\mathrm{P} = \begin{cases} \arctan(s/c), & c \geq 0 \\ \arctan(s/c) + \pi, & c < 0,\ s \geq 0 \\ \arctan(s/c) - \pi, & c < 0,\ s < 0 \end{cases} \qquad \text{[S4]}$$

Supplemental Material, Table S1. Linear model results for birth weight, in grams, showing effect estimates for all covariates, exposed during any trimester, and each trimester individually vs. all trimesters outside fire period.

	exposure in any trimester vs. all trimesters outside fire period		exposure in each trimester vs. all trimesters outside fire period	
	**Effect estimate (change, in g)**	**95% CI**	**Effect estimate (change, in g)**	**95% CI**
**Exposure**				
All trimesters outside fire period	(ref)		(ref)	
Fire period during any trimester	-6.1	(-8.7, -3.5)		
Fire period during first trimester			-3.3	(-7.2, 0.6)
Fire period during second trimester			-9.7	(-14.5, -4.8)
Fire period during third trimester			-7.0	(-11.8, -2.2)
**Fetal sex**				
Male	(ref)		(ref)	
Female	-119.0	(-120.8, -117.1)	-119.0	(-120.8, -117.1)
**Gestational age, weeks**				
37	(ref)		(ref)	
38	143.3	(138.2, 148.4)	143.3	(138.2, 148.4)
39	273.3	(268.7, 278.0)	273.4	(268.7, 278.0)
40	368.9	(364.3, 373.5)	368.9	(364.3, 373.5)
41	441.9	(437.3, 446.6)	441.9	(437.3, 446.6)
42	460.0	(454.8, 465.3)	460.0	(454.8, 465.3)
**Parity**				
1	(ref)		(ref)	
2	90.5	(88.3, 92.8)	90.5	(88.3, 92.8)
3 or more	131.7	(129.3, 134.2)	131.7	(129.3, 134.2)
**Maternal age, years**				
Less than 18	(ref)		(ref)	
18 to 34	46.4	(40.7, 52.0)	46.4	(40.7, 52.0)
35 to 50	71.8	(65.6, 77.9)	71.8	(65.6, 77.9)
**Maternal education**				
Less than high school	-19.3	(-21.9, -16.8)	-19.3	(-21.9, -16.8)
Completed h.s. or equivalent	(ref)		(ref)	
1 to 3 years postsecondary	23.6	(20.8, 26.5)	23.6	(20.8, 26.4)
4 or more years postsecondary	35.3	(32.3, 38.3)	35.3	(32.3, 38.3)
**Maternal race/ethnicity**				
Hispanic	-42.0	(-44.7, -39.3)	-42.0	(-44.7, -39.3)
Non-Hispanic White	(ref)		(ref)	
Non-Hispanic Black	-175.9	(-179.3, -172.5)	-175.9	(-179.3, -172.5)
Non-Hispanic Asian	-166.8	(-171.2, -162.4)	-166.8	(-171.2, -162.4)
Non-Hispanic Multi/Other/Unknown	-31.2	(-47.9, -14.5)	-31.2	(-47.9, -14.5)
**Season conceived**				
January - March	(ref)		(ref)	
April - June	3.4	(0.7, 6.1)	3.9	(1.0, 6.8)
July - September	12.5	(9.8, 15.2)	11.9	(9.0, 14.7)
October - December	3.1	(0.5, 5.7)	2.7	(0.0, 5.4)
**Linear time trend**				
Per year	-6.9	(-7.6, -6.2)	-6.9	(-7.6, -6.2)

Supplemental Material, Table S2. Linear model results for birth weight, in grams, showing effect estimates for all covariates, fire period during each trimester individually vs. all trimesters outside fire period (using cosinor approach rather than seasonal indicator variables).

	Effect estimate (change, in g)	95% CI
**Exposure**		
All trimesters outside fire period	(ref)	
Fire period during first trimester	-3.2	(-5.2, -1.1)
Fire period during second trimester	-11.6	(-14.0, -9.1)
Fire period during third trimester	-6.4	(-8.8, -3.9)
**Fetal sex**		
Male	(ref)	
Female	-118.9	(-119.9, -118.0)
**Gestational age, weeks**		
37	(ref)	
38	143.3	(140.7, 145.9)
39	273.4	(271.0, 275.7)
40	368.9	(366.5, 371.2)
41	441.9	(439.6, 444.3)
42	460.0	(457.4, 462.7)
**Parity**		
1	(ref)	
2	90.5	(89.4, 91.7)
3 or more	131.7	(130.5, 133.0)
**Maternal age, years**		
Less than 18	(ref)	
18 to 34	46.4	(43.5, 49.3)
35 to 50	71.8	(68.7, 74.9)
**Maternal education**		
Less than high school	-19.3	(-20.6, -18.1)
Completed h.s. or equivalent	(ref)	
1 to 3 years postsecondary	23.6	(22.2, 25.1)
4 or more years postsecondary	35.3	(33.8, 36.8)
**Maternal race/ethnicity**		
Hispanic	-42.0	(-43.4, -40.6)
Non-Hispanic White	(ref)	
Non-Hispanic Black	-175.9	(-177.7, -174.2)
Non-Hispanic Asian	-166.8	(-169.0, -164.6)
Non-Hispanic Multi/Other/Unknown	-31.3	(-39.8, -22.7)
**Seasonality (by date of conception**		
Peak-to-peak amplitude	11.6	(7.7, 15.5)
Acrophase (date of maximum birthweight)	Aug 4th	(Jul 29th, Aug7^{th})
**Linear time trend**		
Per year	-6.8	(-7.2, -6.5)

Supplemental Material, Table S3. Linear model results for birth weight, in grams, showing effect estimates for all covariates, fire period during any trimester vs. all trimesters outside fire period, stratified by proximity to monitors with highest PM measures during fire event.

	Proximal to monitors with average PM >=40µg/m^3 during fire period		Proximal to monitors with average PM <40µg/m^3 during fire period	
	**Effect estimate (change, in g)**	**95% CI**	**Effect estimate (change, in g)**	**95% CI**
**Exposure**				
All trimesters outside fire period	(ref)		(ref)	
Fire period during any trimester	-6.6	(-11.0, -2.2)	-5.9	(-9.2, -2.6)
**Fetal sex**				
Male	(ref)		(ref)	
Female	-118.2	(-121.3, -115.1)	-119.4	(-121.7, -117.1)
**Gestational age, weeks**				
37	(ref)		(ref)	
38	149.4	(141.0, 157.89)	139.9	(133.5, 146.3)
39	273.5	(265.8, 281.3)	273.3	(267.5, 279.2)
40	367.1	(359.5, 374.7)	369.7	(363.9, 375.4)
41	436.0	(428.2, 443.8)	445.1	(439.2, 450.9)
42	448.4	(439.7, 457.0)	466.5	(460.0, 473.1)
**Parity**				
1	(ref)		(ref)	
2	88.5	(84.7, 92.2)	91.8	(89.0, 94.6)
3 or more	126.6	(112.6, 130.7)	134.9	(131.8, 138.0)
**Maternal age, years**				
Less than 18	(ref)		(ref)	
18 to 34	48.0	(38.5, 57.5)	45.4	(38.4, 52.5)
35 to 50	72.1	(61.8, 82.4)	70.7	(63.1, 78.4)
**Maternal education**				
Less than high school	-14.5	(-18.7, -10.3)	-22.4	(-25.6, -19.3)
Completed h.s. or equivalent	(ref)		(ref)	
1 to 3 years postsecondary	25.8	(21.3, 30.3)	22.3	(18.7, 26.0)
4 or more years postsecondary	34.8	(30.0, 39.6)	33.8	(30.0, 37.5)
**Maternal race/ethnicity**				
Hispanic	-30.0	(-34.1, -25.9)	-52.0	(-55.5, -48.4)
Non-Hispanic White	(ref)		(ref)	
Non-Hispanic Black	-167.4	(-173.3, -161.6)	-184.0	(-188.2, -179.8)
Non-Hispanic Asian	-159.2	(-166.8, -151.7)	-175.2	(-180.6, -169.8)
Non-Hispanic Multi/Other/Unknown	7.1	(-19.5, 33.7)	-57.7	(-79.2, -36.3)
**Season of conception**				
January–March	(ref)		(ref)	
April–June	3.7	(-0.8, 8.2)	3.2	(-0.2, 6.6)
July–September	11.7	(7.2, 16.1)	12.9	(9.5, 16.3)
October–December	-0.8	(-5.1, 3.5)	5.2	(2.0, 8.5)
**Linear time trend**				
Per year	-7.1	(-8.2, -6.0)	-6.8	(-7.6, -5.9)
